# Still’s Disease Mortality Trends in France, 1979–2016: A Multiple-Cause-of-Death Study

**DOI:** 10.3390/jcm10194544

**Published:** 2021-09-30

**Authors:** Caroline Borciuch, Mathieu Fauvernier, Mathieu Gerfaud-Valentin, Pascal Sève, Yvan Jamilloux

**Affiliations:** 1 Internal Medicine, Croix-Rousse University Hospital, Hospices Civils de Lyon, Université Claude Bernard-Lyon 1, 69004 Lyon, France; caroline.borciuch@chu-lyon.fr (C.B.); ve@chu-lyon.fr (M.G.-V.); pascal.seve@chu-lyon.fr (P.S.); 2 Département de Biostatistique-Bioinformatique, Pôle Santé Publique, Hospices Civils de Lyon, Laboratoire de Biométrie et Biologie Évolutive, Équipe Biostatistique-Santé, Université Lyon 1; CNRS; UMR 5558, 69004 Lyon, France; mathieu.fauvernier@chu-lyon.fr; 3 Research on Healthcare Performance (RESHAPE), INSERM U1290, Université Claude Bernard Lyon 1, 69004 Lyon, France; 4 CIRI (Centre International de Recherche en Infectiologie), Inserm U1111; CNRS, UMR5308; ENS de Lyon, Université Claude Bernard Lyon 1, 69004 Lyon, France; 5 Lyon Immunology Federation (LIFE), 69004 Lyon, France

**Keywords:** adult-onset Still’s disease, systemic-onset juvenile idiopathic arthritis, mortality, epidemiology, hemophagocytic lymphohistiocytosis

## Abstract

Still’s disease (SD) is often considered a benign disease, with low mortality rates. However, few studies have investigated SD mortality and its causes and most of these have been single-center cohort studies. We sought to examine mortality rates and causes of death among French decedents with SD. We performed a multiple-cause-of-death analysis on data collected between 1979 and 2016 by the French Epidemiological Center for the Medical Causes of Death. SD-related mortality rates were calculated and compared with the general population (observed/expected ratios, O/E). A total of 289 death certificates mentioned SD as the underlying cause of death (UCD) (*n* = 154) or as a non-underlying causes of death (NUCD) (*n* = 135). Over the study period, the mean age at death was 55.3 years (vs. 75.5 years in the general population), with differences depending on the period analyzed. The age-standardized mortality rate was 0.13/million person-years and was not different between men and women. When SD was the UCD, the most frequent associated causes were cardiovascular diseases (*n* = 29, 18.8%), infections (*n* = 25, 16.2%), and blood disorders (*n* = 11, 7.1%), including six cases (54%) with macrophage activation syndrome. As compared to the general population, SD decedents aged <45 years were more likely to die from a cardiovascular event (O/E = 3.41, *p* < 0.01); decedents at all ages were more likely to die from infection (O/E = 7.96–13.02, *p* < 0.001).

## 1. Introduction

Still’s disease (SD) encompasses adult-onset Still’s disease (AOSD) and systemic-onset juvenile idiopathic arthritis (sJIA). SD is a rare multisystemic autoinflammatory disorder, characterized by a symptomatic triad associating high spiking fever, arthralgias, and evanescent skin rash [1,2,3]. Biological manifestations might be neutrophilic leukocytosis, hyperferritinemia with collapsed glycosylated ferritin, and abnormal liver function tests [4]. There are several sets of clinical and biological criteria for both AOSD and sJIA that can help clinicians make the diagnosis of SD [5,6,7,8].

Most of the time, SD is seen as a benign disease, with low mortality rates estimated at between 2 and 3% [9,10,11,12]. About one-third of patients develop SD-related complications, such as reactive hemophagocytic lymphohistiocytosis (reHLH, or macrophage activation syndrome), pulmonary hypertension, or myocarditis [13,14,15,16]. A recent analysis of a large US secondary care database found a mortality rate of 2.6%. Reactive HLH (1.7%), disseminated intravascular coagulation (1.1%), and thrombotic thrombocytopenic purpura (0.4%) complicated the hospital course and increased the fatality rate [12].

Studies investigating SD mortality are scarce and most of these focus on specific complications or are single-center cohort studies, limiting the generalization of the results. Multiple-cause-of-death (MCOD) analysis provides comprehensive data at a country level with complete coverage of all deaths over selected periods of time and has been used successfully to study mortality in rare chronic diseases such as inflammatory or autoimmune conditions [17,18,19]. We therefore took advantage of the systematic recording of death certificates by the French Epidemiological Center for the Medical Causes of Death (*Centre d’épidémiologie sur les causes médicales de décès*, CepiDC) to evaluate SD-related mortality using MCOD analysis and compare it to that of the general population.

## 2. Patients and Methods

### 2.1. Data Source and Retrieval

For this MCOD analysis, we used the same methodology as described previously [17,18,19]. Briefly, the CepiDC has collected and logged all death certificates issued in France since 1979. Anonymous data are freely available to researchers in France. French death certificates comply with the World Health Organization (WHO) standards and consist of two parts. Part I lists the “diseases related to the morbid process leading to death”, defined as the underlying cause of death (UCD). Part II lists the “other significant conditions contributing to death, but not related to the disease or condition causing it”, defined as non-underlying causes of death (NUCDs). Diagnoses are coded according to the International Classification of Diseases (ICD; version 9 between 1979 and 1999, or version 10 after 2000). The use of these anonymized data does not require ethics committee approval. We obtained data from all death certificates that mentioned the codes 7142 and 7143 in ICD9, or M06.1 and M08.2 in ICD10, corresponding to sJIA and AOSD, either as a UCD or NUCD between 1979 and 2016. In addition, sex, place of death (hospital, private clinic, retirement home, public place), and age at death (10-year age groups) were also collected. For the general population, the mean age at death was obtained from national statistics (https://www.insee.fr/fr/statistiques/4204054?sommaire=4204068, accessed on 24 August 2021).

### 2.2. MCOD Analysis

The number of death certificates that listed SD as the UCD and the number of death certificates that listed SD as a UCD or NUCD, whatever its place on the death certificate, were considered in the MCOD analyses. For each death certificate that listed SD as the UCD, the NUCDs were examined. For each death certificate that listed SD as an NUCD, the UCD was investigated and the observed/expected (O/E) ratios were calculated for three age groups. For each UCD, the O/E ratio was calculated based on the proportional mortality rate for the same UCD within the general French population (available online at http://cepidc-data.inserm.fr/inserm/html/index2.htm, accessed on 24 August 2021).

### 2.3. Statistical Analysis

Quantitative variables were described using means and standard deviations, while qualitative variables were described as frequencies and percentages of each modality. Observed mortality rates were calculated using the corresponding person-years from the French population (period 1979–2016), according to sex, age, and year. Age-standardized mortality rates (aSMR; per 1 million person-years) were then calculated by a direct method, using the age distribution of the 2006 general population of France as reference. In order to consider the effects of sex, year, and age on the mortality rate, we fitted a generalized additive model (GAM) with a Poisson distribution. The non-linear effects of age and year were modeled using penalized cubic splines [20]. The effect of sex was considered constant whatever the age or year. Differences were considered statistically significant when *p* < 0.05. R software version 3.5.2 (www.r-project.org/foundation/, accessed on 24 August 2021)was used for the statistical analysis; the mgcv package was used for the penalized Poisson model.

## 3. Results

### 3.1. Characteristics of SD Decedents

Among the 20,460,583 death certificates recorded in France by the CepiDC, between 1979 and 2016, 289 certificates reported a diagnosis of SD; thus, the mean annual number of related deaths was 7.81/year. When death was directly linked to SD (UCD, *n* = 154), 62% of the decedents had AOSD and 38% had sJIA. Twenty-two certificates mentioned more than one NUCD.

There were 120 men and 169 women; the men-to-women sex ratio of decedents was 0.71 (Table 1). Over the study period, the mean age at death was 55.3 years in SD patients, with differences depending on the period analyzed; between 1979 and 1999, the mean age at death was 52.5 years, while between 1999 and 2016 it was 58.2 years. Over the same period, the mean age at death was 75.5 years in the general population.

**Figure 1:**
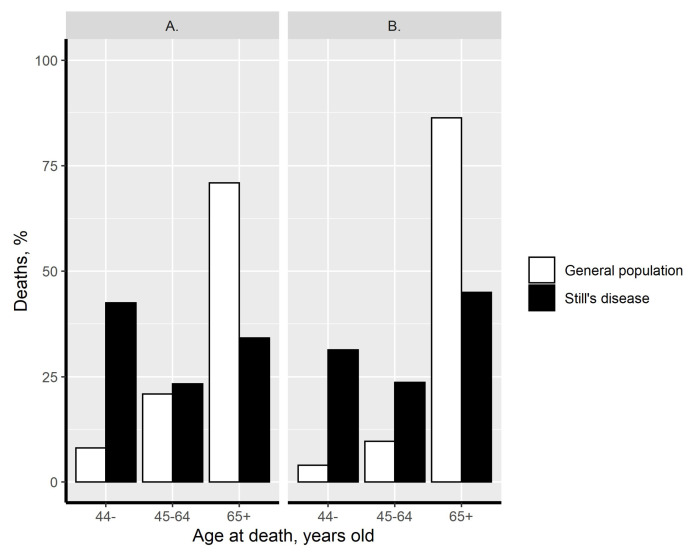
Distribution of deaths according to age. The distribution of Still’s disease-related deaths (black bars) and that in the general population (white bars) according to age is presented among men (A) and women (B).

Compared to all-cause death in the general population, a greater proportion of SD-related deaths occurred before the age of 44 years for men and women, and this proportion was also greater in women aged between 45 and 64 years (Figure 1). This premature death was not observed in men aged between 45 and 64 years. The distribution of SJIA-related deaths and that in the general population according to age is shown in Figure S1.

### 3.2. Age-Standardized Mortality Rate

**Figure 2:**
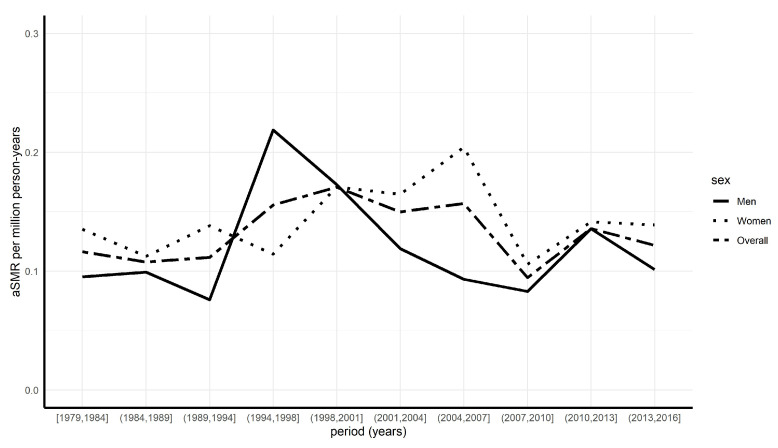
Evolution of the age-standardized mortality rate (aSMR) during the period 1979 to 2016 according to periods of time and modeling. The time periods were set in a way to ensure a homogeneous distribution of cases in the intervals.

The overall age-standardized mortality rate (aSMR) among SD decedents was 0.13 per million person-years (Table 1). This rate was variable over the study period with an increase during the 1990s, followed by a progressive decrease and stabilization (Figure 2).

**Figure 3:**
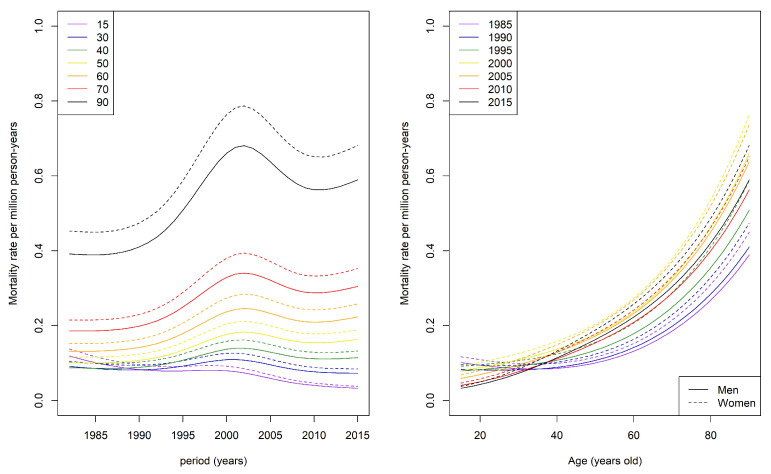
Predicted mortality rate per 1 million person-years using the generalized additive model (taking into account sex and year of death, **left panel**) and predicted mortality rate per 1 million person-years using the generalized additive model (taking into account sex and age at death, **right panel**).

Taking into account sex, year of death, and age on the mortality rate, a GAM found that the mortality rate increased from the early 1990s, peaked around 2002, and decreased, reaching a plateau for decedents before the age of 50 years, whereas it still increased for decedents after the age of 50 years (Figure 3, left panel). This suggests that, over time, SD decedents were older. This increase, dependent on the study period, is illustrated in the right panel of Figure 3.

The risk of death in women was multiplied by 1.16, but this was not statistically significant (*p* = 0.229).

### 3.3. MCOD Analysis

Still’s disease was listed as the UCD on 154/289 (53%) death certificates and as an NUCD on the remaining 135/289. SD notification as the UCD was higher in the younger group of decedents; SD was reported as the UCD in 67% (*n* = 70) of cases aged <45 years at death, vs. 40% (*n* = 27) of cases aged 45–64 years at death, vs. 49% (*n* = 57) among those aged >65 years at death. Over the years, notification of SD as the UCD slightly but progressively decreased, whereas notification as an NUCD remained stable. Before 2000, SD notification as the UCD was higher in decedents aged <44 than in older decedents. After 2000, SD notification as the UCD was higher in decedents aged >65 than in younger decedents. The same trends were observed for the declarations of SD as an NUCD.

When SD was the UCD (*n* = 154), the three most frequently determined NUCDs were: cardiovascular diseases (*n* = 35, 22.7%), infections (*n* = 27, 17.5%), and blood disorders (*n* = 12, 7.7%), which were reactive hemophagocytic syndromes (i.e., macrophage activation syndromes) in six cases (50%) (Table 2 and Table S1). There were no significant differences between men and women. A total of 50 (32.5%) death certificates mentioned SD as the UCD without further details. Between 2000 and 2016, a second NUCD was identified in 15 cases, including six cases of cardiovascular diseases, three respiratory diseases, three solid malignant neoplasms, one lymphoma, and two infections.

When SD was listed as an NUCD (*n* = 135), the main UCDs were cardiovascular diseases (*n* = 37, 27.4%) (Table 3). Infections were the second most frequently reported UCD (*n* = 31, 23.0%), while the third was solid malignancies (*n* = 18, 13.3%).

In the whole cohort (*n* = 289), the main reported causes of death associated with SD were cardiovascular diseases (*n* = 72) and infections (*n* = 58). Pulmonary infections were the most frequently reported infections (*n* = 33). Pneumocystis pneumonia was reported in two cases.

When a cardiovascular disease was listed as the UCD or an NUCD, the main diagnoses in patients who died before the age of 44 (*n* = 23) were cardiac insufficiency (*n* = 5, 21%), pericarditis (*n* = 4, 17%), pulmonary embolism (*n* = 2, 8.6%), or arrhythmia (*n* = 2, 8.6%). In patients who died after the age of 65 from cardiovascular disease (*n* = 34), 82% of deaths were attributed to two causes: arterial events (myocardial infarction or stroke, *n* = 14, 41%) and cardiac insufficiency (*n* = 14, 41%). Pericarditis was mentioned in four cases (one UCD, three NUCDs; all aged between 15 and 44), while endocarditis and myocarditis were mentioned in one case each. Pulmonary hypertension was notified as an NUCD in three cases (one death before age 24 and two after age 55).

Blood disorders were reported in 27 patients (9.3%). Macrophage activation syndrome (MAS) was reported in 15 of these cases (55%). In six additional cases, an MAS could be suspected but was not reported as such. In five cases with MAS, there was an associated mention of disseminated intravascular coagulation (DIC). MAS notification was homogenously distributed among the age categories. There were two notifications of thrombotic microangiopathy (not associated with MAS notification). Two lymphomas were reported (one as the UCD, one as an NUCD), along with one report of acute myeloid leukemia. Amyloidosis was reported in eight (2.7%) patients, with 63% of the cases reported before the age of 44 and 87% of the cases reported before 2002. In 6/8 cases, only amyloidosis and SD were reported on the death certificate; in the remaining cases, there was one case of arrhythmia and one case of cardiac hypertrophy.

There were six reports of pulmonary fibrosis (three as UCD, three as NUCD); all were over 45 years of age and 4/6 (75%) were over 65 years. There were also two reports of associated giant cell arteritis and one sarcoidosis (all as NUCDs).

### 3.4. Observed/Expected Ratios

There was a significant excess of mortality due to cardiovascular diseases compared to the general population when age at death was <45 years of age (O/E = 3.41, *p* < 0.01) (Table 4).

Infections caused a significant excess of mortality in all categories of age, with the highest O/E ratio observed in decedents between age 45 and 64 (O/E = 13.02, *p* < 0.001).

A significant excess of mortality due to blood disorders was observed for cases who died before 65 years of age (O/E = 7.29–8.17, *p* < 0.001).

There was no significant difference in O/E ratios in mortality due to cancer in all age categories.

## 4. Discussion

Our study is the first MCOD analysis of Still’s disease in Europe. Over the study period, the mean reported age at death was 55.3 years, while it was 75.5 years in the general population. The main explanation for this result is that SD (sJIA or AOSD) preferentially affects children and young adults. This is further illustrated by the comparison with the general population, which shows that the proportion of people who died because of SD was much higher among those under 44 years of age. However, in the whole cohort, 64% of the cases were over 45 years of age at the time of death. This indicates that the analysis of death certificates has probably captured cases of prolonged SD (chronic forms, which could also be more severe forms), when most epidemiological studies show the age at onset. Indeed, a retrospective US study of 154 AOSD patients hospitalized between 2009 and 2013 revealed a mean age at death of 62.4 years [12]. In another study from Korea, the average age of expired patients was 49.2 years [21]. Over the study period, the age-standardized mortality rate varied but ultimately remained the same between 1979 and 2016. There is probably no single explanation for this result; however, recent therapeutic advances (notably the widespread use of interleukin-1 blockers) rather suggest that this result is related to better identification/reporting of the disease.

The sample size of our study may seem quite limited, but SD is a rare disorder with very low mortality [3,12]. Moreover, MCOD analyses provide comprehensive data at a national level. Thus, the results of such studies have to be interpreted as broad signals rather than sharp and specific analyses.

For example, we found that patients mostly died from cardiovascular diseases. This specific excess mortality was only observed in young decedents, indicating that SD or its treatment could be considered as a cardiovascular risk factor. Chronic systemic inflammatory response and the prolonged use of steroids are associated with an increased risk of cardiovascular disease and atherosclerosis [22,23]. However, such an observation is rather rare in the literature dealing with SD, as most studies focus on cardiovascular mortality induced by acute and life-threatening complications such as myocarditis, endocarditis, or the risk of tamponade due to pericarditis [13,14,15,16,24,25]. Only one nationwide study from Poland reported that the most common comorbidities in 1050 AOSD patients were circulatory system diseases (14%) [26]. Thus, in addition to routine care, close cardiovascular monitoring, together with screening and treatment of classical risk factors, should be part of the management of SD patients.

The second most common cause of death associated with SD was infection, which again has been poorly reported in the literature. A few studies have shown that infections are the first cause of death in SD [21,27,28]; all were reported as complications of immunosuppressive treatment. Because ruling out infection is mandatory in the differential diagnosis of SD, the literature mostly deals with infections as part of the diagnosis workup. In addition, studies are more likely to focus on SD-specific complications, such as MAS. However, the reader should bear in mind that the main triggers for MAS (termed secondary hemophagocytic lymphohistiocytosis when it is not associated with an underlying rheumatic disorder) are infections [29,30,31]. In the context of SD, it is suggested that the onset of MAS may be co-triggered by infection, which should always be sought prior to the initiation of heavy immunosuppressive therapy [32,33]. The findings of the present study should remind physicians to use the least immunosuppressive treatment possible and optimize the control of infectious risk (prophylaxis, vaccination).

In our study, fifteen deaths (5%) were directly or indirectly attributable to MAS. This finding contrasts with an earlier report, which indicated that up to 63% of SD decedents die of refractory MAS [30]. While MAS may have been underestimated in the 1970s and the 1980s (as it was a newly described and little known condition), such an extreme number arises from a small cohort (*n* = 19) and is not repetitively alleged [10,12,21]. The frequency of MAS as a complication of SD is probably underestimated but varies from 2.8 to 14.3% [10,30,34,35,36,37]. MAS may evolve to uncontrolled coagulopathy with features of DIC, as observed in one-third of the cases associated with MAS in the present series. DIC has been associated with a 30-fold increase in the risk of in-hospital death in SD [12]. Early treatment of MAS is thus required to avoid this evolution.

In two additional cases, thrombotic microangiopathy was mentioned as an underlying cause of death. Thrombotic microangiopathy is an exceptional complication of SD, with less than 50 cases reported so far [14]. This result highlights the ability of MCOD analysis to detect rare complications of a disease due to its comprehensive nature.

Overall, a significant excess mortality due to blood disorders was observed in young decedents with O/E ratios ranging from 7.29 to 8.17. These results confirm the relationship between SD and hematological complications, along with their severity.

While considered as uncommon, amyloidosis has long been reported as a possible complication driving mortality in SD [38,39]. In this context, it is related to the deposition in tissues of serum amyloid A protein secondary to persistent or recurrent inflammation. We found eight (2.8%) decedents with an amyloidosis notification either as the UCD or an NUCD. Recently, a systematic review of the literature (1971–2018) estimated the prevalence of AA amyloidosis in AOSD to be 0.88% [40]. In this article, Delplanque et al. described 19 patients with a case fatality rate of 21%. This result was half that of Smith et al. in 1968 [38]. They also nicely presented the case reports of such an association in the literature over the decades. They showed that, while incidence and mortality reports increased before the decade 2000–2010, they then decreased in the following decades. Consistent with this picture, most death certificates mentioning amyloidosis were dated before 2002 in our study. These findings suggest the improved management of SD and better prevention and recognition of inflammatory amyloidosis.

While pulmonary fibrosis is a known complication of SD, the association of giant cell arteritis or sarcoidosis with SD is unusual. Unfortunately, by analyzing death certificates, it is not possible to know whether these were linked disorders, misdiagnoses, or incidental associations. As SD diagnosis is based on the exclusion of multiple differential diagnoses, some signs or symptoms associated with vasculitis may overlap with those of SD. In addition, lymph node enlargement may also challenge the diagnosis [41]. Very recently, a new entity with vasculitis, skin, and joint involvement has been added to the list of differential diagnoses of SD, namely VEXAS syndrome [42]. Some of the VEXAS patients in the original cohort had a diagnosis of SD, while others had a diagnosis of giant cell arteritis. The somewhat intriguing results of our study remind us how difficult the diagnosis of SD remains.

Interestingly, there was no increase in O/E ratios for solid or hematological malignancies, which can be interpreted as a reassuring signal for SD patients.

The limitations of this study are mainly related to the method used. In particular, the accuracy of the completion of death certificates cannot be guaranteed. As in other studies that used a similar methodology, it can be assumed that previous or resolved SD (i.e., monocyclic forms), or quiescent/non-active SD, was not reported on the death certificates. As a result, the most severe cases of SD may be over-represented. However, this limitation is also present in retrospective cohort studies, which usually include cases referred to tertiary centers. This bias also affects inpatient cohorts.

The small sample size is probably not sufficient to provide accurate estimates, but the fact that the cases are unselected and captured longitudinally does allow for some representativeness, particularly with regard to SD-specific mortality.

Finally, the differentiation between the UCD and NUCD in decedents with Still’s disease might have been difficult. However, in SD, life-threatening complications are fairly well identified.

Despite these limitations, this study highlights some specific medical priorities in the management of SD patients, particularly regarding cardiovascular and infectious risks. It also confirms the important association with hematological disorders such as MAS, a major factor in mortality. Finally, this study tends to confirm the decline of AA amyloidosis over time, especially since the early 2000s.

## 5. Conclusions

The prognosis of SD has improved over the last two decades. Apart from the widely described specific causes of death, such as MAS or DIC, physicians should pay special attention to frequent and preventable causes of death, including cardiovascular disease and infections, focusing on their prevention. MAS can occur at any age and appears to be the most common hematological cause of death in SD.

## Figures and Tables

**Table 1:** Absolute number of deaths associated with Still’s disease in France between 1979 and 2016, and according to year groups.

Period of Time	(1979, 1984)	(1984, 1989)	(1989, 1994)	(1994, 1998)	(1998, 2001)	(2001, 2004)	(2004, 2007)	(2007, 2010)	(2010, 2013)	(2013, 2016)	Total
**All SD-related deaths** **(men/women)**	38 (15/23)	29 (13/16)	31 (10/21)	35 (21/14)	30 (14/16)	26 (10/16)	29 (8/21)	18 (7/11)	27 (12/15)	26 (10/16)	289 (120/169)
**Age**											
<45 years	25	15	12	15	10	5	6	5	5	6	104
45–64 years	7	5	5	8	6	10	7	5	6	9	68
≥65 years	6	9	14	12	14	11	16	8	16	11	117
**Cause of death (men/women)**											
SD = UCD	24 (10/14)	18 (9/9)	18 (5/13)	20 (13/7)	15 (9/6)	13 (7/6)	13 (2/11)	9 (3/6)	12 (5/7)	12 (4/8)	154 (67/87)
SD = NUCD	14 (5/9)	11 (4/7)	13 (5/8)	15 (8/7)	15 (5/10)	13 (3/10)	16 (6/10)	9 (4/5)	15 (7/8)	14 (6/8)	135 (53/82)
**SD listed as the UCD** **Age**											
<45 years	17	12	6	11	6	4	6	1	3	4	70
45–64 years	2	1	2	4	3	4	3	2	3	3	27
≥65 years	5	5	10	5	6	5	4	6	6	5	57
**SD listed as an NUCD** **Age**											
<45 years	8	3	6	4	4	1	0	4	2	2	34
45–64 years	5	4	3	4	3	6	4	3	3	6	41
≥65 years	1	4	4	7	8	6	12	2	10	6	60
**Age-standardized mortality rate** *											
Overall	0.12	0.11	0.11	0.16	0.17	0.15	0.16	0.09	0.14	0.12	0.13
Men	0.1	0.1	0.08	0.22	0.17	0.12	0.09	0.08	0.14	0.1	0.12
Women	0.14	0.11	0.14	0.11	0.17	0.16	0.2	0.11	0.14	0.14	0.14

* per million person-years; SD: Still’s disease, UCD: underlying cause of death, NUCD: non-underlying cause of death. The time periods were set in a way to ensure a homogeneous distribution of cases in the intervals.

**Table 2:** Non-underlying causes of death when SD was listed as the underlying cause of death, for the period 1979–2016.

	Men	Women	Total
	*n* = 67	*n* = 87	*n* = 154
Infections	9 (13.4%)	16 (18.4%)	25 (16.2%)
Cardiovascular diseases	13 (19.4%)	16 (18.4%)	29 (18.8%)
Gastrointestinal diseases	0 (0)	6 (6.9%)	6 (3.9%)
Respiratory diseases	6 (9.0%)	4 (4.6%)	10 (6.5%)
Blood disorders	7 (10.4%)	4 (4.6%)	11 (7.1%)
Solid malignant neoplasms	0 (0)	1 (1.1%)	1 (0.6%)
Unclassified above	12 (17.9%)	10 (11.5%)	22 (14.3%)
No NUCD notification	20 (29.9%)	30 (34.5%)	50 (32.5%)

**Table 3:** Underlying causes of death when SD was listed as a non-underlying cause of death, for the period 1979–2016.

	Men	Women	Total
	*n* = 53	*n* = 82	*n* = 135
Cardiovascular diseases	12 (22.6%)	25 (30.5%)	37 (27.4%)
Infections	14 (26.4%)	17 (20.7%)	31 (23.0%)
Solid malignant neoplasms	8 (15.1%)	10 (12.2%)	18 (13.3%)
Blood disorders	8 (15.1%)	7 (8.5%)	15 (11.1%)
Gastrointestinal diseases	0 (0.0%)	4 (4.9%)	4 (3.0%)
Respiratory diseases	2 (3.8%)	2 (2.4%)	4 (3.0%)
Others	9 (17.0%)	17 (20.7%)	26 (19.3%)

**Table 4:** Observed/expected (O/E) ratios for underlying causes of death when SD was a non-underlying cause of death *.

	<44 Years Old		45–64 Years Old		>65 Years Old	
	O/E Ratio (95% CI)	*p*	O/E Ratio (95% CI)	*p*	O/E Ratio (95% CI)	*p*
Cardiovascular diseases	3.41 (1.56–6.48)	<0.01	0.68 (0.22–1.59)	0.446	1.1 (0.7–1.65)	0.665
Infectious diseases	8.78 (4.54–15.34)	<0.001	13.02 (5.95–24.71)	<0.001	7.96 (3.82–14.64)	<0.001
Blood disorders	7.29 (2.67–15.86)	<0.001	8.17 (3.53–16.11)	<0.001	0.81 (0.02–4.5)	0.877
Solid malignant neoplasms	NA	NA	0.55 (0.26–1.01)	0.078	0.59 (0.25–1.16)	0.174

* For each death certificate that listed Still’s disease as a non-underlying cause of death (NUCD), the underlying causes of death (UCDs) were investigated and the observed number of deaths in relation to the expected number of deaths (O/E ratio) was calculated. 95%CI = 95% confidence interval.

## Data Availability

Data are available on reasonable request from the corresponding author.

## Supplementary Materials

The following are available online at https://www.mdpi.com/article/10.3390/jcm10194544/s1, Figure S1: Distribution of deaths according to age in individuals aged under 35. The distribution of systemic-onset juvenile idiopathic arthritis (SJIA)-related deaths (black bars) and that in the general population (white bars) according to age is presented, Table S1: Second non-underlying causes of death when SD was listed as the underlying cause of death, for the period 1979–2016.
