# Supplementary material for: Still’s Disease Mortality Trends in France, 1979–2016: A Multiple-Cause-of-Death Study
Source: J Clin Med. 2021 Sep 30;10(19):4544. doi: 10.3390/jcm10194544 (PMC8509657; doi:10.3390/jcm10194544)
Supplement: Supplementary file 1 [file jcm-10-04544-s001.zip › jcm-1373691-supplementary.pdf]

Supplementary material

related to: **Still's disease mortality trends in France, 1979-2016: a multiple-cause-of-death study**

by: Caroline Borciuch[1], Mathieu Fauvernier[2], Mathieu Gerfaud-Valentin[1], Pascal Sève[1,3], and Yvan Jamilloux[1,4,5,*]

Figure S1. Distribution of deaths according to age in individuals aged under 35. The distribution of systemic-onset juvenile idiopathic arthritis (SJIA)-related deaths (black bars) and that in the general population (white bars) according to age is presented.

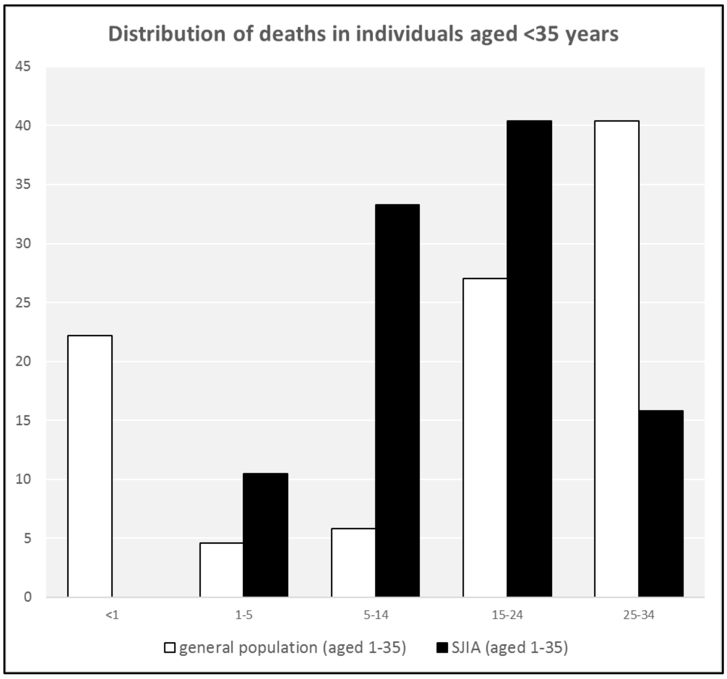

Table S1. Second non-underlying causes of death when SD was listed as the underlying cause of death, for the period 1979-2016.

	**Men**<br>n=67	**Women**<br>n=87	**Total**<br>n=154
Infections	1 (1.5%)	1 (1.1%)	2 (1.3%)
Cardiovascular diseases	3 (4.5%)	3 (3.4%)	6 (3.9%)
Gastrointestinal diseases	0 (0)	0 (0)	6 (0)
Respiratory diseases	2 (3.0%)	1 (1.1%)	3 (1.9%)
Blood disorders	0 (0.0%)	1 (1.1%)	1 (0.6%)
Solid malignant neoplasms	2 (3.0%)	1 (1.1%)	3 (1.9%)
Unclassified above	12 (17.9%)	10 (11.5%)	22 (14.3%)
No 2nd NUCD notification	59 (88.1%)	80 (92.0%)	139 (90.3%)
